# Effects of different exhalation valves on CO_2_ rebreathing and ventilator performance during noninvasive ventilation

**DOI:** 10.3389/fmed.2025.1538280

**Published:** 2025-07-23

**Authors:** Xinyu Li, Bing Dai, Chengguang Zhou, Haijia Hou, Shengchen Wang, Xiangrui Li, Hongwen Zhao, Wei Wang, Wei Tan

**Affiliations:** ^1^Department of Respiratory and Critical Care Medicine, The First Affiliated Hospital of China Medical University, Shenyang, China; ^2^Shenyang RMS Medical Tech Co., Ltd., Shenyang, China

**Keywords:** noninvasive ventilation, exhalation valves, CO_2_ rebreathing, ventilator performance, plateau exhalation

## Abstract

**Background:**

Noninvasive ventilation (NIV) is widely used to improve oxygenation and reduce carbon dioxide (CO_2_) retention in patients with respiratory failure. However, it remains unclear whether different types of exhalation valves affect CO_2_ rebreathing and ventilator performance during NIV.

**Methods:**

Three noninvasive ventilators (V60, Flexo, and Stellar150) with single-limb circuits and four different exhalation valves (single-arch, whisper swivel, plateau exhalation, and vented mask valves) were separately connected in series to a lung simulator. CO_2_ gas was injected from the simulated lung outlet, maintaining the end-expiratory CO_2_ (PetCO_2_) at 80 mmHg. Both the CO_2_ rebreathing volume (CO_2REB_) and the parameters displayed on the lung simulator and ventilator were recorded under each condition.

**Results:**

The mean CO_2REB_ values of the four aforementioned valves were 18.51 ± 2.87, 18.25 ± 2.73, 17.78 ± 2.98, and 14.26 ± 0.92 mL/breath, respectively, with no significant differences among the first three types but all significantly higher than that of the mask valve (all *p* < 0.0001, rate of difference > 10%). Except with the V60 ventilator, some ventilator performance parameters (triggering and control performance) were significantly lower for the plateau valve than for the others, the rate of difference in tidal volume (V_T_) between the ventilator and the simulated lung exceeded 10% for all exhalation valves (all *p* < 0.01).

**Conclusion:**

Mask valves showed significantly lower CO_2_ rebreathing than circuit-located valves (single-arch, whisper swivel, and plateau exhalation) in this NIV bench study. The different valves influenced ventilator performance differently, particularly the plateau valve. These findings necessitate further clinical validation *in vivo*.

## Introduction

Noninvasive ventilation (NIV) is extensively used among patients with acute and chronic respiratory failure, reducing the intubation rate, the risk of respiratory failure after extubation, hospital length of stay, and mortality (1–5). In certain patient populations, such as those experiencing an exacerbation of chronic obstructive pulmonary disease (COPD), NIV is considered first-line therapy and the standard of care (1–3).

NIV is delivered via a noninvasive ventilator with a single limb, utilizing an exhalation port that directs exhaled gasses from the breathing circuit to the atmosphere. Currently, four types of exhalation valve are available for clinical practice: the single-arch, plateau, whisper swivel, and vented mask (integrated leak port within the mask) valves (6). The characteristics of such valves including design features that allow controlled amounts of air to escape as “intentional leaks” vary greatly (7), influencing rebreathing risk and carbon dioxide retention, particularly in hypercapnic respiratory failure. Recent clinical data (8) suggested that insufficient CO_2_ clearance may be associated with worse medium-term outcomes, highlighting the importance of CO_2_ washout during inpatient NIV use. Besides, the characteristics of exhalation valves affecting the ability of a ventilator to generate pressure and thus its performance; this is particularly true of plateau exhalation valves (7). However, it remains unclear whether commercial exhalation valves differ in their effects on CO_2_ rebreathing and ventilator performance when used with different ventilators, and the optimal standards for exhalation valve selection have yet to be identified. We therefore employed bench methodologies to systematically quantify valve performance mechanisms, establishing a mechanistic basis for subsequent clinical validation.

In the present bench study, we applied the simulated lung model to comprehensively compare CO_2_ rebreathing among the four types of valve in combination with three noninvasive ventilators. We also evaluated the impact of valve type on ventilator performance, including triggering, control, and monitoring parameters.

## Materials and methods

### Bench model setup

The Active Servo Lung simulation system (ASL5000, Ingmar, Pittsburgh, PA) is a precise breathing simulator containing a piston that moves inside a cylinder, capable of simulating breathing patterns of patients with COPD. The following parameters adopted from previous literature were applied to the simulated COPD lung model: compliance of 60 mL/cmH_2_O, inspiratory resistance of 10 cmH_2_O/L/s, expiratory resistance of 15 cmH_2_O/L/s, and maximum inspiratory pressure drop of −8 cmH_2_O. To simulate the profile of the negative pressure produced by respiratory muscles, 5% of the respiratory cycle time was set to active inspiration, 3% to an end-inspiratory hold, and 15% to return pressure to baseline (9–13). Breathing frequency was set at 10, 15, and 25 breaths/min, respectively.

Three noninvasive ventilators were used: the V60 (Philips, Carlsbad, United States), Flexo (Curative Medical, Jiangsu, China), and Stellar150 (ResMed, Saint Priest, France) ventilators. Both the Flexo and Stellar150 ventilators were used in S (Spontaneous) mode with an inspiratory pressure airway pressure (IPAP) and expiratory positive airway pressure (EPAP) of 12/4 and 16/4 cmH_2_O, respectively. The other settings were as follows: inspiratory trigger sensitivity (Isens) of 2, expiratory trigger sensitivity (Esens) of 2, and inspiratory slope (Islop) of 2 for the Flexo ventilator; and a trigger of high, switch of high, rise of 150, and decline of 10 for the Stellar150. The V60 ventilator was used in S/T (Spontaneous/Timed) mode with the lowest respiratory rate (4 breaths/min), as there is no S mode. The pressure settings were the same as above, with the following additional settings: inspiratory time (Ti) of 0.9, rise time of 1, and trigger of automatic. If miss-triggering occurred, the trigger parameter was readjusted to avoid this.

**Figure 1:**
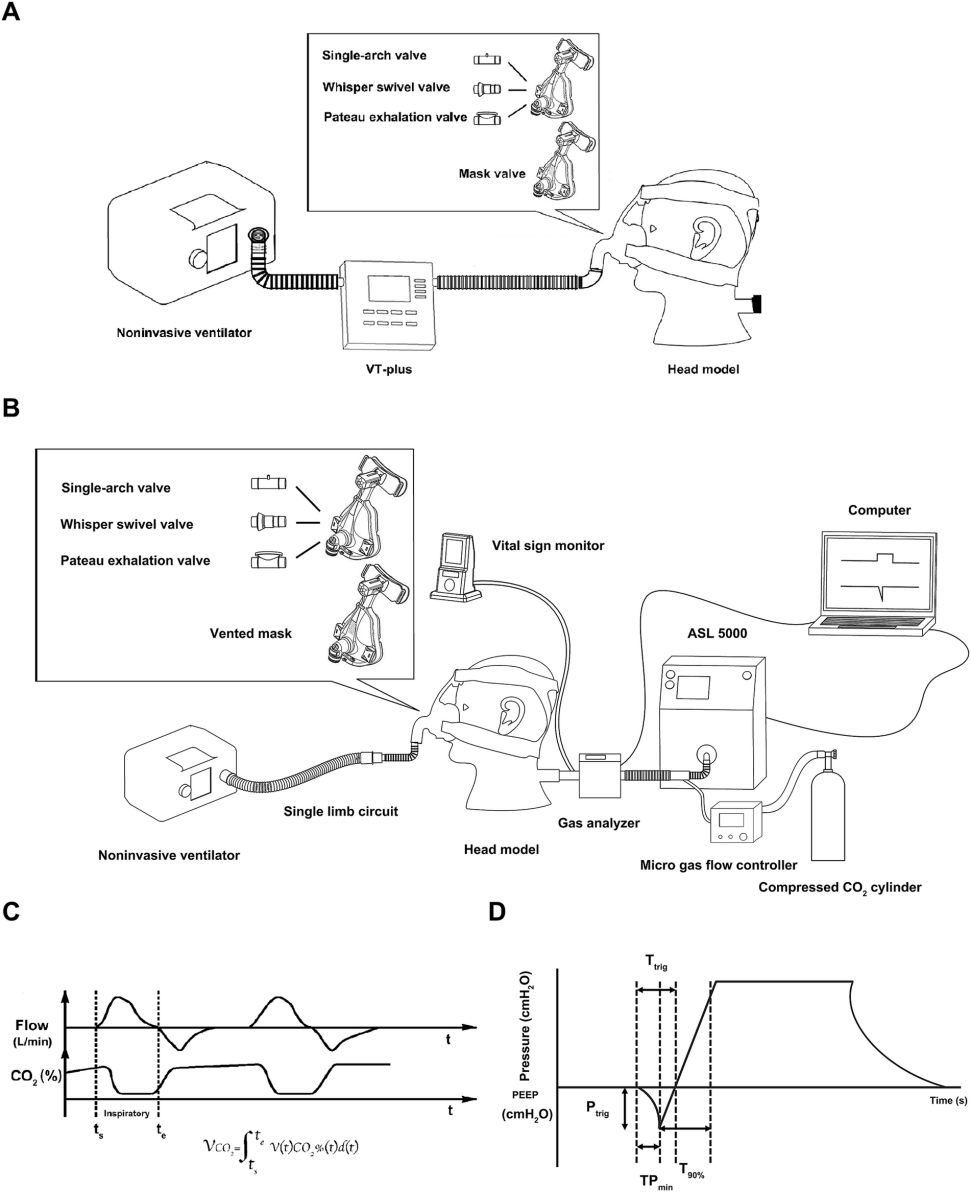
Illustration of the experiment. **(A)** Measuring the intentional leak of the exhalation valves, **(B)** schematic diagram of experimental setup, **(C)** time curve of flow and CO_2_ concentration and the equation, **(D)** graphic explanation of the ventilator parameters.

Two oronasal masks (first- and second-generation), with identical inner volumes and shape, were used from the same manufacturer (Bestfit™ I/II; Curative, Santa Clara, California). The first-generation mask, without an exhalation valve, was separately connected to a single-arch valve, whisper swivel valve, and plateau exhalation valve (all from Respironics, Andover, MA). The second-generation mask has an exhalation port integrated into the mask itself, located above the nose bridge. To measure leak of the exhalation valve (Figure 1A), the VT Plus airflow analyzer (Fluke Biomedical, Everett, WA) was connected in series in the single-limb breathing circuit. The mask was fixed to the face of an adult-sized model head equipped with a mouth port and a headband. To avoid unintentional leaks, plasticine was used to seal any spaces around the interface between the interface and the head model. The leakage of each valve was measured at continuous positive airway pressures (CPAPs) of 4, 12, and 20 cmH_2_O.

The bench model is diagrammed in Figure 1B. The noninvasive ventilator was connected to the simulated lung through a single-limb breathing circuit behind the model head, which was connected one of the four valves. A micro gas flow controller (YJ-700CF, kongxin instrument company, Guangxi, China) was connected to a compressed CO_2_ cylinder (0.3 kpa), and CO_2_ was injected from the simulated lung outlet. The partial pressure of end-tidal carbon dioxide (PetCO_2_) was monitored using a vital sign monitor (NTID, newtech medical, Shenzheng, China), which was situated behind the model head to maintain PetCO_2_ at 80 mmHg. A self-made gas analyzer, integrating flow rate and CO_2_ sensors, was located between the model and the lung simulator.

### Data acquisition

Inspiratory flow was continuously monitored with a flow sensor (OOM109/OOM109-LF2, EnviteC, Wismar/Honeywell, Morristown, NJ) at a sampling frequency of 30 ms. The inspiratory phase was identified according to the flow waveform. We designed software that multiplied the CO_2_ concentration by the inspiratory flow at each sampling point during the inspiratory phase. The delivered CO_2_ volume was calculated using the equation shown in Figure 1C.

When the noninvasive ventilator and CO_2_ ran stable for more than 5 min, ventilator parameters were recorded for 10 consecutive respiratory cycles (Figure 1D). The parameters included triggering performance, including time to trigger (Ttrig, in milliseconds [ms]), time from the beginning of the lung simulator’s inspiratory effort to the lowest airway pressure needed to trigger the ventilator (TPmin), and the magnitude of airway pressure drop needed to trigger (Ptrig); control performance, dividing into time to achieve 90% of the inspiratory target during inspiration (T90%, in ms), inspiratory time (Ti), peak inspiratory pressure (PIP), peak inspiratory flow (PIF), mean inspiratory flow (MIF), and positive end expiratory pressure (PEEP); and tidal volume (V_T_) parameters including actual V_T_ (V_T-L_, that displayed on the simulated lung) and monitored V_T_ (V_T-V_, that displayed on the ventilator).

### Statistical analysis

All statistical analyses were performed using GraphPad Prism (GraphPad Software, San Diego, CA). Data are expressed as means ± standard deviations. Differences between parameters displayed on the ventilator and lung simulator were calculated as follows: rate of difference = (the parameters displayed on the ventilator – those on the lung simulator) / the parameters displayed on the lung simulator. A paired *t*-test was used to assess these differences. One-way analysis of variance (ANOVA) was used to compare the influence of different exhalation valves on CO_2_ rebreathing and ventilator performance under the same condition. Changes were considered clinically significant only when the rate of difference exceeded 10% and the differences were statistically significant at *p* < 0.05 (9–13).

## Results

### Leakage

**Figure 2:**
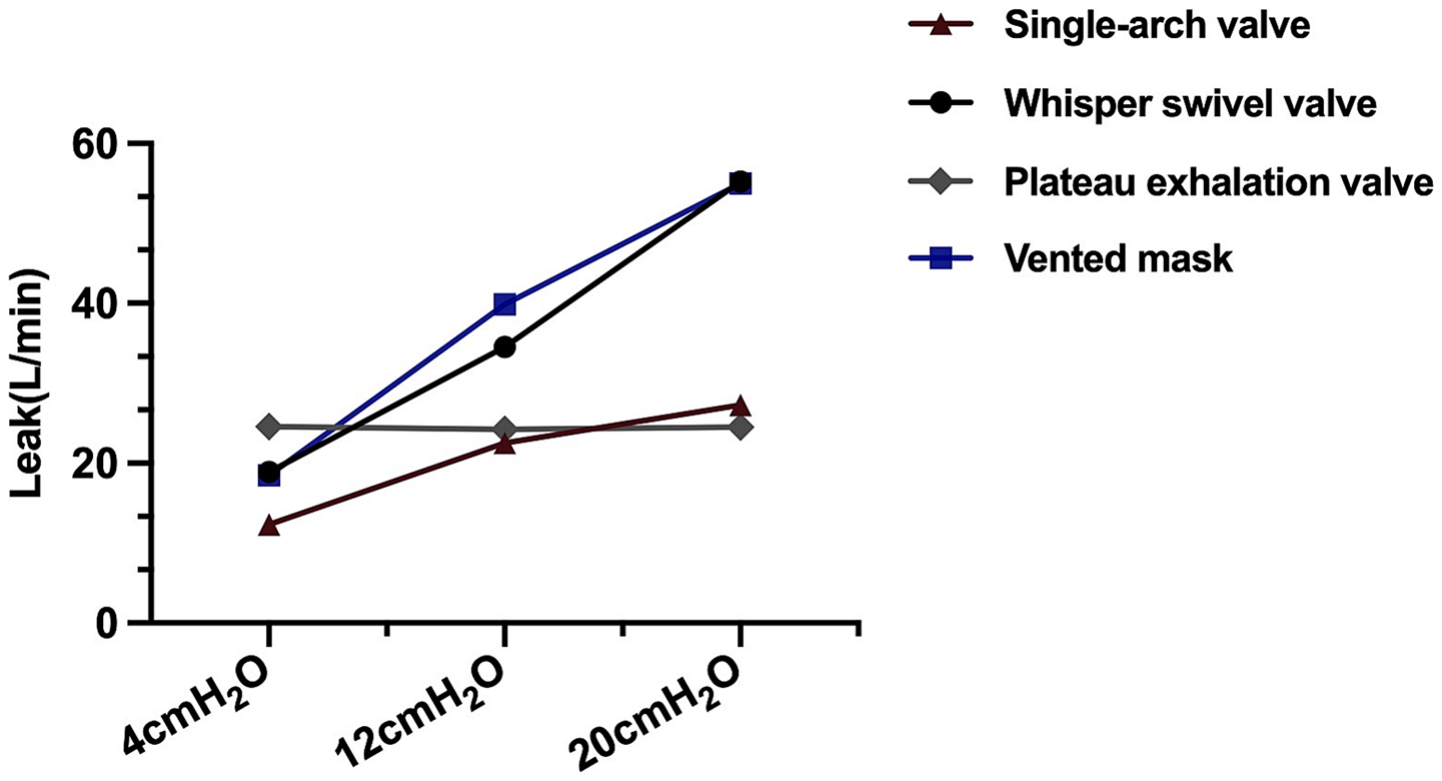
The leakage of different exhalation valves.

The leakages of different exhalation valves are shown in Figure 2. Those of the single-arch, whisper swivel, and mask valves increased linearly with increasing CPAP. With CPAP as the independent variable and leakage as the dependent variable, the general linear equations for the valves were y = 1.08x + 8.54 (*R*^2^ = 0.99, *p* < 0.01) y = 1.64x + 12.93 (*R*^2^ = 0.99, *p* < 0.01), and y = 2.28x + 10.40 (*R*^2^ = 0.99, *p* < 0.01), respectively. In contrast, the plateau exhalation valve exhibited a relatively constant leakage at different CPAP levels (24.44 ± 0.9 L/min) (Figure 2).

### CO_2_ rebreathing

**Figure 3:**
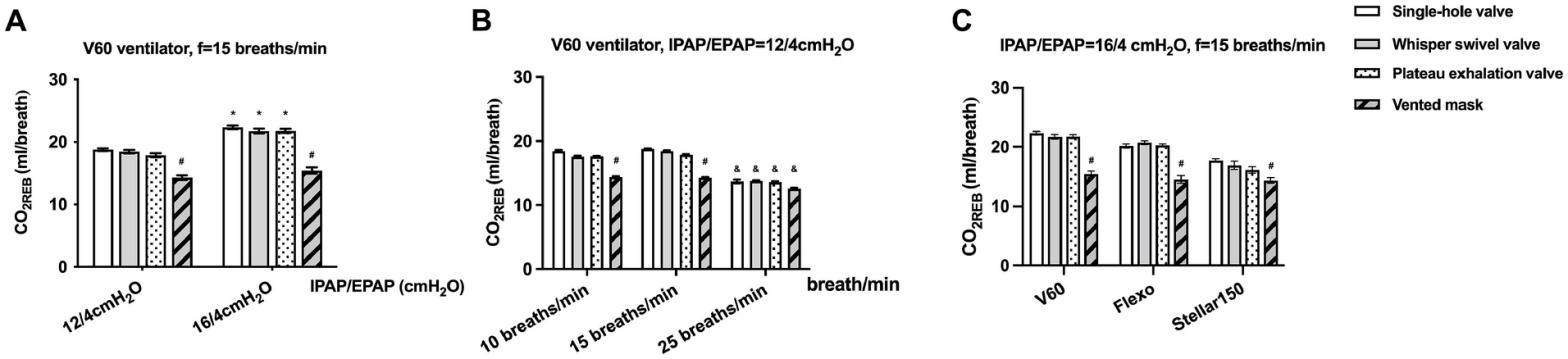
The CO_2REB_ of different exhalation valves under different conditions. **(A)** Comparison under different pressure settings (IPAP/EPAP = 12/4 and 16/4 cmH2O); **(B)** Comparison at different breathing frequencies (10, 15, and 25 breaths/min); **(C)** Comparison across different ventilator types (V60, Flexo, and Stellar150). * Significantly higher than IPAP = 12cmH_2_O (*p* < 0.01), # significantly lower than other exhalation valves (*p* < 0.01), & significantly lower than *f* = 10/15 breaths/min (*p* < 0.01).

The mean CO_2REB_ values under different ventilator settings for the single-arch, whisper swivel, plateau, and mask valves were 18.51 ± 2.87, 18.25 ± 2.73, 17.78 ± 2.98, and 14.26 ± 0.92 mL/breath, respectively. There were no significant differences among the first three valve types, all of which were significantly higher than that of the mask valve (all *p* < 0.0001, rate of difference > 10%). Similar results were observed at different pressure settings (IPAP/EPAP = 12/4 and 16/4 cmH_2_O) (Figure 3A) and breathing frequencies (10 and 15, but not at 25, breaths/min) (Figure 3B), and for different types of ventilator (V60, Flexo, and Stellar150) (Figure 3C).

### Ventilator performance

**Figure 4:**
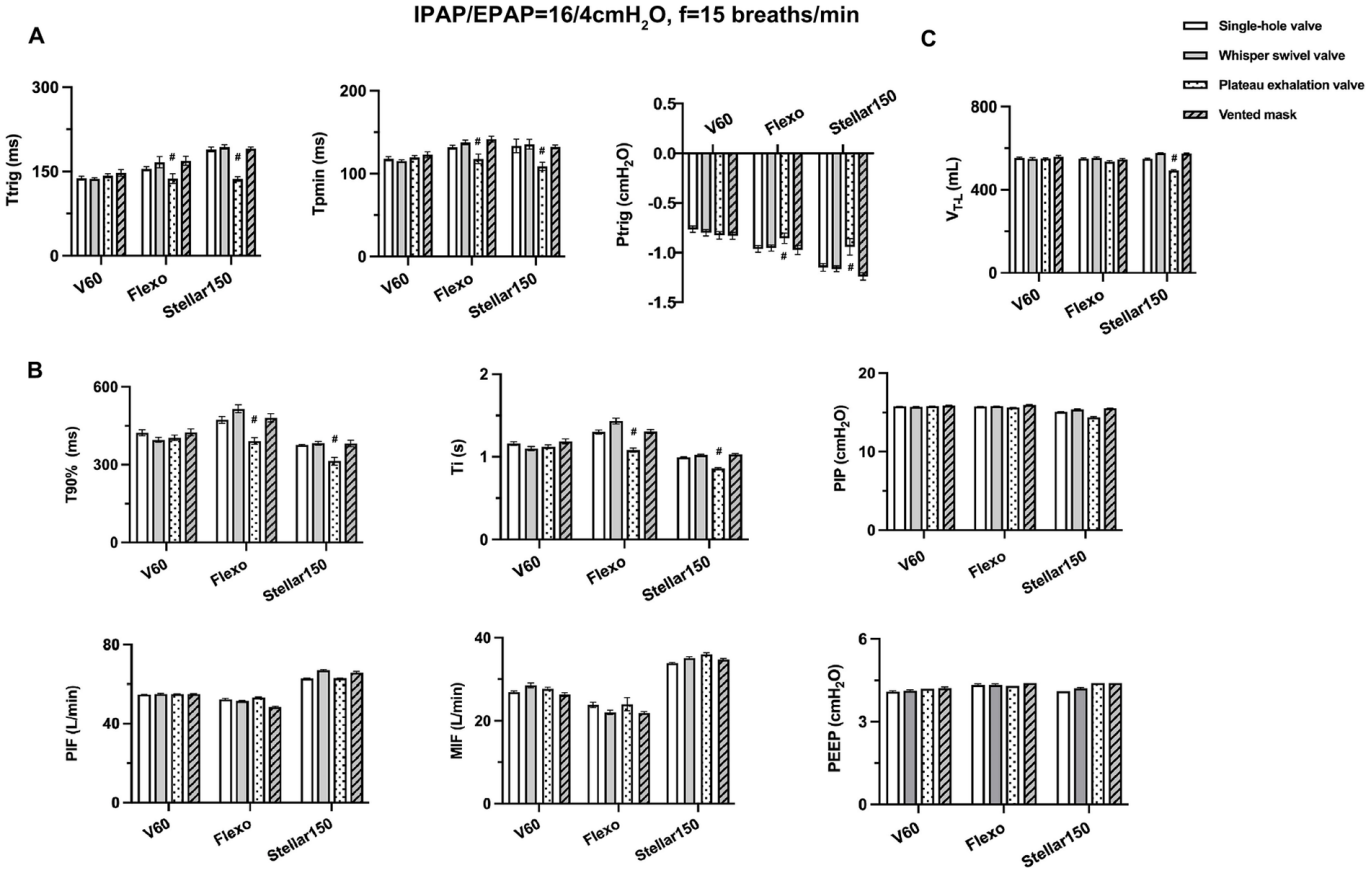
Effects of different exhalation valves on ventilator performance. **(A)** Triggering parameters (Ttrig, Tpmin and Ptrig); **(B)** control parameters (T90%, Ti, PIP, PIF, MIF and PEEP); **(C)** tidal volume displayed on the lung simulator (V_T-L_). # significantly lower than other exhalation valves (*p* < 0.01).

The effects of each valve on ventilator performance varied greatly, particularly for the plateau valve. In regard to triggering performance (Ttrig, Tpmin, Ptrig), values were significantly lower for the plateau valve versus all others (all *p* < 0.01, rate of difference > 10%), except for the V60 ventilator (Figure 4A). Regarding control performance, T90% and Ti were the lowest when using the plateau valve with the Flexo and Stellar150 ventilators (all *p* < 0.01, rate of difference > 10%); there were no significant differences in PIP, PIF, MIF, or PEEP among the valves (Figure 4B). Finally, in terms of V_T-L_, the value was significantly lower when using the plateau valve with the Stellar150 ventilator than any other combination (all *p* < 0.01, rate of difference > 10%) (Figure 4C).

Except for the V60 ventilator, the rate of difference in V_T_ between the ventilator and the simulated lung exceeded 10% for all exhalation valve types. The rate of difference in PIP remained below 10% under all conditions (Table 1).

**Table 1:** The difference rate of tidal volume (Vt, mL) and peak inspiratory pressure (PIP, cmH₂O) between the ventilator and simulated lung with various exhalation valve types.

	V60			Flexo			Stellar150		
	V_t -L_	V_t -V_	the difference rate,%	V_t -L_	V_t -V_	the difference rate,%	V_t -L_	V_t -V_	the difference rate,%
The difference rate of Vt (mL) between ventilator and simulated lung	553.53±4.34	595.40±2.84	7.57±0.79	549.70±4.43	819.0±14.86	49.0±2.87	548.58±4.01	611.15±5.35	11.41±0.44
	547.76±6.99	592.90±3.03	8.25±1.34	553.09±4.85	826.7±6.25	49.48±1.67	577.02±2.61	631.4±3.72	24.59±0.91
	550.40±3.40	599.10±2.73	8.85±0.86	535.27±5.18	829.1±10.51	54.91±2.47	492.14±3.45	394.63±103.5	-19.7±0.81
	558.56±6.49	600.50±4.06	7.52±1.36	546.06±4.83	932.9±4.04	70.86±1.70	573 56±2 66	702.29±0.8	22.44±0.5
	V60			Flexo			Stellar150		
	PIP _-L_	PIP_-V_	the difference rate,%	PIP _-L_	PIP_-V_	the difference rate,%	PIP_-L_	PIP_-V_	the difference rate,%
The difference rate of PIP (cmH_2_O) between ventilator and simulated lung	15.78±0.02	16.0	1.38±0.12	15.77±0.02	16.0	1.58±0.13	15.10±0.04	16.0	6.11±0.32
	15.73±0.03	16.0	1.69±0.22	15.82±0.02	16.0	1.30±0.14	15.39±0.06	16.0	4.00±0.40
	15.79±0.02	16.0	1.36±0.13	15.60±0.02	16.0	2.31±0.16	14.39±0.10	16.0	6.23±0.74
	15.88±0.04	16.0	0.73±0.29	15.96±0.02	16.0	0.21±0.15	15.50±0.03	16.0	3.20±0.20

## Discussion

We comprehensively assessed the effects of four types of exhalation valve on CO_2_ rebreathing and ventilator performance under different NIV settings and ventilators. Although no significant differences in CO_2_ rebreathing were observed among the single-arch, whisper swivel, and plateau valves, all showed significantly higher rebreathing volumes than the mask valve. Additionally, except for the V60 ventilator, some ventilator performance parameters were significantly lower for the plateau valve than for the others, and the rate of difference in V_T_ between the ventilator and the simulated lung with all exhalation valves exceeded 10%.

NIV can be delivered via a single-limb circuit, requiring a valve to enable exhalation. Clinically, four types of such valve are commonly used, depending on preference and equipment availability. However, their structural and functional characteristics differ. Previous study has indicated that leakage from the single-arch, whisper swivel, and mask valves increases with pressure, whereas the plateau valve maintains relatively constant leakage across different pressure levels due to its unique silicon diaphragm structure (7). Our study corroborates these findings, and further comprehensively analyzed their impact on CO_2_ rebreathing and ventilator performance.

Both bench and clinical studies (14–18) have demonstrated that CO_2_ may not be adequately cleared during NIV, for an additional rebreathing process occurred and exhaled gasses constituted a fraction of the delivered tidal volume. Various factors—including patient-associated characteristics (e.g., baseline CO_2_ levels, spontaneous breathing patterns), ventilator settings (e.g., IPAP, EPAP), and interface-related elements (e.g., exhalation valve type and position)—have been reported to affect CO_2_ rebreathing during NIV (15–18). The simulated COPD lung model, which replicates key pathophysiological features of hypercapnic COPD such as increased dead space, has been widely used in previous bench studies (9, 11) to evaluate CO_2_ clearance efficacy. Similarly, maintaining a simulated PetCO_2_ of 80 mmHg—consistent with our previous approach (9)—provides a standardized hypercapnic condition for assessing the impact of different exhalation valves on rebreathing. As in prior methodology (15), the calculated CO_2_ rebreathing represented the total rebreathing volume from the ventilator circuit, mask, and upper airway dead space of the mannequin, and not solely the CO_2_ rebreathed from the circuit. Besides, previous studies also suggested that CO_2_ rebreathing can be minimized by using an EPAP of up to 8 cmH_2_O or an IPAP of up to 20 cmH_2_O (16). Therefore, to eliminate ventilator parameter influences while accounting for clinical constraints (specifically, the 4 cmH_2_O minimum EPAP setting on most devices), we applied IPAP levels of 12 or 16 cmH_2_O with a fixed EPAP of 4 cmH_2_O in this bench study. This design enables isolated investigation of exhalation valve effects on CO_2_ rebreathing within an *in vitro* COPD model.

We found that CO_2_ rebreathing with the mask valve was significantly lower than with the single-arch, whisper swivel, and plateau valves. This aligns with Schettino et al. (15), who demonstrated that exhalation port position influences CO_2_ rebreathing. Compared to circuit-located leak ports, the mask-integrated exhalation port (as in the Facial-MEP design) enhances residual gas washout by fresh airflow, reducing dead space ventilation and rebreathing volume. Meanwhile, our previous work confirms substantial design-dependent variation in CO_2_ rebreathing among oronasal masks with integrated ports, with mean values ranging 11.01–16.78 mL (9). Notably, all integrated-port masks exhibited lower rebreathing volumes than single-arch, whisper swivel, and plateau valves in this study.

Among the circuit-located exhalation ports, the plateau valve is generally believed to facilitate lower CO_2_ rebreathing due to its unique controlled leakage mechanism (16). However, no significant differences in CO_2_ rebreathing were observed among the single-arch, plateau, and whisper swivel valves in our study. This aligns with Hill et al.’s *in vivo* findings (19), where plateau valves showed no clinical advantage over whisper swivel valves in long-term NIV patients. This null effect likely relates to leakage characteristics. Except the minimum EPAP (4 cmH_2_O), plateau valves demonstrated equivalent leakage to whisper swivel and single-arch valves at both 12 and 20 cmH_2_O (Figure 2). Consequently, at our tested IPAP levels (12/16 cmH_2_O) with fixed EPAP (4 cmH_2_O), plateau valves conferred no additional rebreathing reduction. Notably, Ferguson and Gilmartin (16) observed superior performance of plateau valves at EPAP = 0 cmH_2_O, with valve differences disappearing at higher EPAP levels. Thus, at clinically relevant pressures (>4 cmH_2_O EPAP), rebreathing differences among these circuit-located valves become negligible. Further integrated bench-to-bedside studies are warranted to define valve selection criteria across therapeutic pressure ranges.

In terms of ventilator performance, we found that the plateau valve differed from the others. Triggering parameters (Ttrig, Tpmin, Ptrig) and control parameters (T90%, Ti) were significantly lower than for the others across the tested ventilators, except for the V60 ventilator. This may be due to the constant leakage of the plateau valve, and there is a greater leakage during the expiratory phase when EPAP is relatively low. This greater leakage may cause the ventilator to mistakenly perceive that the patient starting to inhale, thereby prematurely triggering the ventilator to deliver gas to the patient, resulting in a decrease in triggering parameters. Additionally, the relatively low leakage with the plateau valve at higher IPAP settings (16 cmH_2_O) may cause the ventilator to switch to expiration earlier, resulting in shorter inspiratory times and reduced tidal volumes. Therefore, when selecting exhalation valves in clinical practice, it is crucial to consider their impact on triggering and control performance during NIV.

Furthermore, we found that the rate of difference in V_T_ between the ventilator and the simulated lung exceeded 10% for all exhalation valve types when using the Flexo and Stellar150 ventilators but not the V60 ventilator. During NIV, the monitored tidal volume displayed on the ventilator is an estimated value. Luján et al. (20) reported that different valve types have different leakage, which can affect the accuracy of ventilator parameter monitoring during NIV. To be able to display accurate tidal and minute volumes, the ventilator must know the intentional leak characteristics of the specific mask/patient interface and exhalation port. With V60 ventilator, select the desired exhalation port type or/and run an exhalation port test is needed before the ventilator starts working. We previously found that tidal volume monitoring was more accurate after leak tests when using certain industrial masks (13). These findings suggest that exhalation valves may not be interchangeable among ventilators with single-limb circuits. If interchangeable, leak tests of the valves from different manufacturers may be required.

Our *in vitro* findings yield some clinical and research implications. Consistent with prior studies, mask-integrated valve demonstrated significantly lower CO_2_ rebreathing than circuit-located valves (single-arch, whisper swivel, plateau) during NIV, aligning with our previous work confirming superior CO_2_ clearance across oronasal mask valves. Crucially, the plateau valve—despite its controlled-leak mechanism—showed no rebreathing reduction at clinically relevant settings, this provides actionable guidance for optimizing NIV trial protocols and clinical implementation. Furthermore, valve-dependent performance variations revealed some imperatives: rigorous monitoring of ventilator parameters (trigger sensitivity, control, and ventilation monitoring) during exhalation valves selection; development of ventilators with real-time intentional leak monitoring to optimize ventilator performance; and future studies also need to validate these findings across different patient populations (e.g., restrictive lung diseases) through bench-to-bedside investigations.

Several methodological limitations warrant consideration. This *in vitro* analysis employed a simulated COPD model with fixed hypercapnia (PetCO_2_ 80 mmHg) and restricted ventilator pressure settings, thereby failing to capture physiological variations in respiratory patterns, metabolic CO_2_ fluctuations, or compensatory mechanisms in chronic respiratory failure. The restriction to 12/4 and 16/4 cmH_2_O pressure settings—while clinically relevant—precludes evaluation across the full therapeutic spectrum, and the absence of human variables (e.g., airway humidity, spontaneous breathing variability) limits clinical extrapolation. Though providing rigorously controlled conditions for quantifying CO_2_ rebreathing dynamics, these constraints necessitate: (1) expanded pathophysiological models (e.g., restrictive disease); (2) systematic valve evaluation across incremental pressures (e.g., IPAP 8–25 cmH₂O; EPAP 4–12 cmH_2_O); and (3) prioritized clinical validation measuring ventilator performance parameters, and gas exchange efficacy in target populations.

## Conclusion

Our bench study demonstrated that different exhalation valves significantly affect CO_2_ rebreathing and ventilator performance during NIV. CO_2_ rebreathing was significantly lower with the mask valve than with the single-arch, whisper swivel, and plateau valves during NIV. Except for the V60 ventilator, ventilator performance parameters of the plateau valve differed from those of the other valves; additionally, the rate of difference in V_T_ between the ventilator and the simulated lung exceeded 10% for all exhalation valves. Further clinical studies are needed to validate these results *in vivo*, taking into account patient variability in respiratory mechanics, ventilator settings, and CO_2_ production.

## Data Availability

The raw data supporting the conclusions of this article will be made available by the authors, without undue reservation.
